# Machine learning for the prediction of bone metastasis in patients with newly diagnosed thyroid cancer

**DOI:** 10.1002/cam4.3776

**Published:** 2021-03-12

**Authors:** Wen‐Cai Liu, Zhi‐Qiang Li, Zhi‐Wen Luo, Wei‐Jie Liao, Zhi‐Li Liu, Jia‐Ming Liu

**Affiliations:** ^1^ Department of Orthopaedic Surgery The First Affiliated Hospital of Nanchang University Nanchang PR China; ^2^ The First Clinical Medical College of Nanchang University Nanchang PR China; ^3^ Institute of Spine and Spinal Cord Nanchang University Nanchang PR China

**Keywords:** bone metastasis, machine learning, random forest, SEER, thyroid cancer

## Abstract

**Objectives:**

This study aimed to establish a machine learning prediction model that can be used to predict bone metastasis (BM) in patients with newly diagnosed thyroid cancer (TC).

**Methods:**

Demographic and clinicopathologic variables of TC patients in the Surveillance, Epidemiology, and End Results database from 2010 to 2016 were retrospectively analyzed. On this basis, we developed a random forest (RF) algorithm model based on machine‐learning. The area under receiver operating characteristic curve (AUC), accuracy score, recall rate, and specificity are used to evaluate and compare the prediction performance of the RF model and the other model.

**Results:**

A total of 17,138 patients were included in the study, with 166 (0.97%) developed bone metastases. Grade, T stage, histology, race, sex, age, and N stage were the important prediction features of BM. The RF model has better predictive performance than the other model (AUC: 0.917, accuracy: 0.904, recall rate: 0.833, and specificity: 0.905).

**Conclusions:**

The RF model constructed in this study could accurately predict bone metastases in TC patients, which may provide clinicians with more personalized clinical decision‐making recommendations. Machine learning technology has the potential to improve the development of BM prediction models in TC patients.

## Graphical abstract

We developed a random forest prediction model for bone metastases in thyroid cancer (TC) patients. This facilitates personalized diagnosis and refined clinical decision making for bone metastases in TC patients.

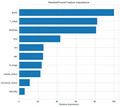

## 1 INTRODUCTION

Thyroid cancer (TC) is the most common endocrine malignant tumor, and its incidence has increased sharply all over the world in recent decades.^1^, ^2^ Because of its biological characteristics and response to effective treatment, the patients with TC have an excellent long‐term prognosis.^3^, ^4^ However, if a TC patient has distant metastasis (DM), the overall prognosis will deteriorate significantly.^5^, ^6^, ^7^

According to reports, approximately 4% of TC patients will develop BM.^8^ The 5‐year survival rate of TC patients who develop BM is 61%, and the 10‐year survival rate is 27%.^9^ The majority of TC metastases are asymptomatic and are detected only during systemic surveillance or systemic metastatic examination of malignant thyroid nodules. Because of the low incidence and asymptomatic nature of BM, testing for BM is often overlooked during the initial diagnosis of a patient with TC. The current detection method is mainly bone scanning, however, due to the defects of high cost, radiation damage, and low sensitivity to micrometastases focus.^10^ Patients’ bone scanning are recommended only in the presence of suspicious skeletal‐related events (SRE), and it has been reported that the median time to develop SRE is 5 months after bone metastasis (BM).^6^ By then, many TC patients may miss out on the best treatment opportunities because they may have developed an advanced disease or multiple metastases. Machine‐learning (ML) technology makes it possible to infer important connections between data items from disparate data sets otherwise these data items will be difficult to correlate.^11^, ^12^ Today, the sheer volume and complexity of medical data make the use of ML in diagnosing disease and predicting clinical outcomes promising. ML has been used in clinical settings and have demonstrated greater accuracy than conventional methods.^13^, ^14^

Therefore, we aim to establish a machine learning‐based predictive model for predicting BM occurrence of patients with TC. This study may provide clinicians with more personalized clinical decision making and allocate health resources more appropriately.

## 2 MATERIALS AND METHODS

### 2.1 Study population

**FIGURE 1:**
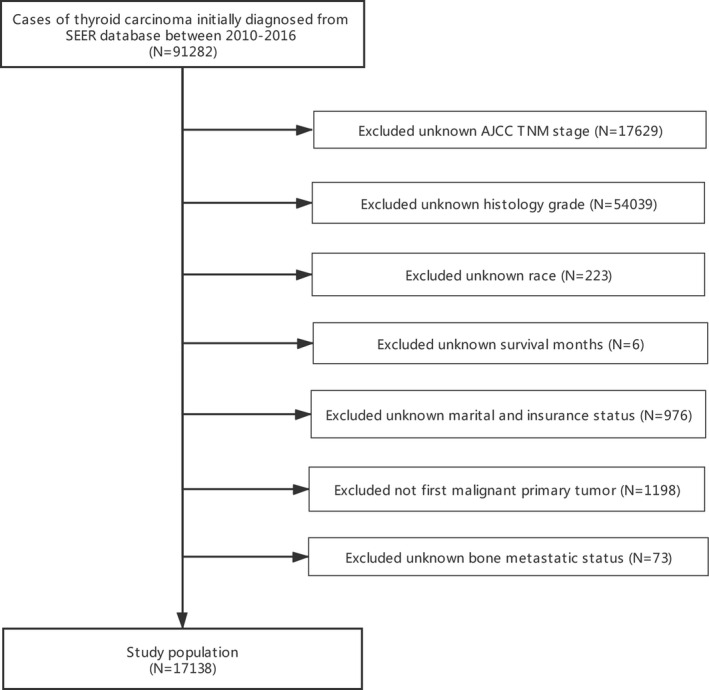
Flow diagram of the study population selected from the Surveillance, Epidemiology, and End Results (SEER) database. Based on the inclusion and exclusion criteria, 17,138 patients were included in this study

This study was derived from the Surveillance, Epidemiology, and End Results (SEER) database. Patient data were downloaded from the “SEER 18 Regs Research Data + Hurricane Katrina Impacted Louisiana Cases (1973–2016)” by using SEER*stat 8.3.8 software. The study was limited to the period between 2010 and 2016, as information on metastasis at the site of interest was only available in 2010 and later. Also the criteria of exclusion are as follows: (1) unknown information of T stage, N stage, race, grade, insurance status, marital status, and bone metastatic status; (2) TC is not the first tumor. Meanwhile, the patient selection procedure is displayed in Figure 1. The seventh edition of the AJCC TNM staging system was used as the basis for staging the cases included in the study.

### 2.2 Data selection

In this study, a total of 10 population and clinicopathological variables were included. Population variables include sex, age, race, marital status, and insurance status. Clinicopathology variables include laterality, grade, histology, T stage, and N stage. Histological types are classified into four categories according to IDO‐O‐3 Codes: “anaplastic thyroid cancer (ATC) 8020.8021.8030.8032.”; “follicular thyroid cancer (FTC) 8330. 8331. 8335.”; “medullary thyroid cancer (MTC) 8510.”; and “papillary thyroid cancer (PTC) 8340.8341.8342.8344.8260.” All methods were carried out according to the SEER database's relevant guidelines.

### 2.3 Model establishment

**FIGURE 2:**
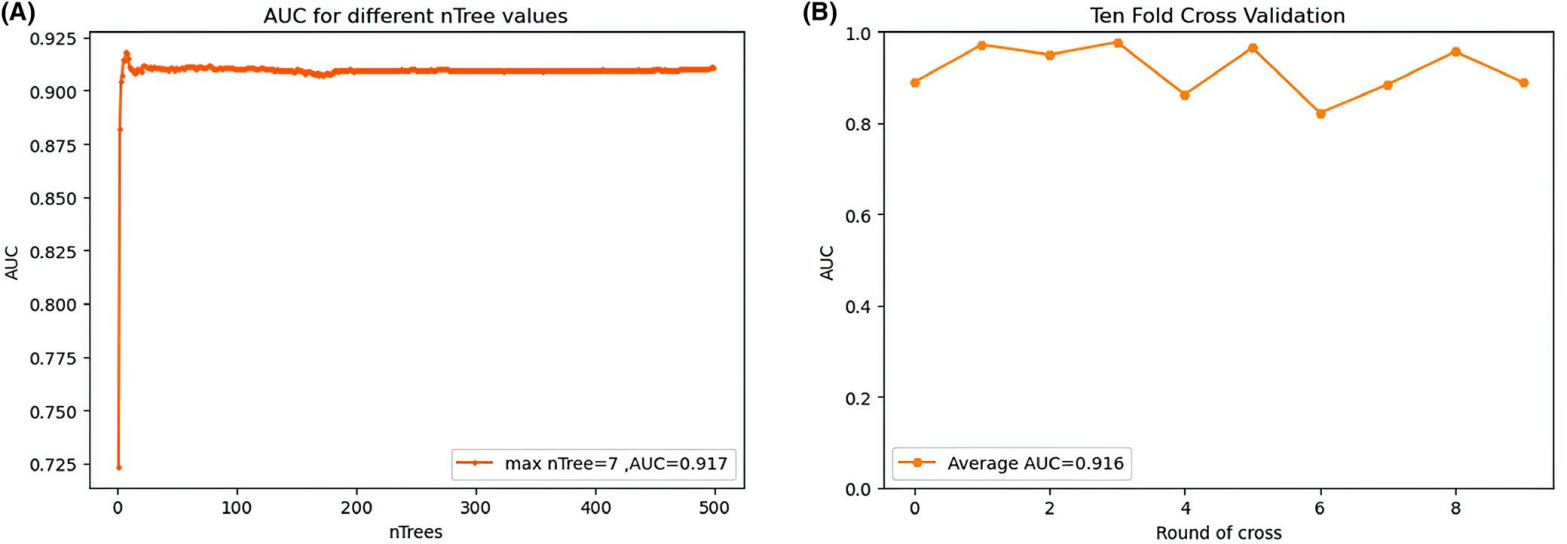
(A) Area under the curve (AUC) values for ntree values from 1 iterates to 500 in the improved random forest model. (B) Ten‐fold cross‐validation of the improved random forest model

All statistical analysis in the study was performed with R (version 3.6.8, R Foundation for Statistical Computing) and Python (version 3.7, Python Software Foundation). All variables were tested for Pearson correlation with each other, and the results are presented with a heat map (Figure 2). All patients were randomly divided into training set and test set at 7:3 (Table 1). The chi‐square test was used to analyze the differences between the training and test sets. The training set was used to establish a random forest (RF) model and a multivariate logistic regression (LR) model, and the test set was applied to evaluate them. For RF, it builds Bagging integration based on decision tree (DT), and further introduces random attribute selection in the training process of DT. Figuratively speaking, it is to build many DTs to form a “forest” of DTs, and make decisions through the voting of multiple trees. This method can effectively improve the classification accuracy of new samples.^15^ The randomness of the RF is reflected in the fact that the training samples for each tree are random, and the splitting properties of each node in the tree are randomly selected. With these two random factors, the RF does not over‐fit even if no pruning is performed on each DT. At first, we used the number of trees in a RF (ntree = 500) to build the model. For multivariate LR, we use an enter variable selection method to establish the model. Area under the receiver operating characteristic curve (AUC), accuracy score, recall rate, and specificity were applied to compare the prediction power of two models.

**TABLE 1:** Clinical and pathological characteristics of training set and test set

Variables	Training set		Test set		*p* value
	NBM (*n* = 11,885) (%)	BM (*n* = 112) (%)	NBM (*n* = 5087) (%)	BM (*n* = 54) (%)	
Age					0.498
<50	5779 (48.6)	20 (17.9)	2510 (49.3)	4 (7.4)	
≥50	6106 (51.4)	92 (82.1)	2577 (50.7)	50 (92.6)	
Sex					0.988
Male	2996 (25.2)	53 (47.3)	1281 (25.2)	25 (46.3)	
Female	8889 (74.8)	59 (52.7)	3806 (74.8)	29 (53.7)	
Race					0.386
Black	859 (7.2)	19 (17.0)	383 (7.5)	10 (18.5)	
Other	1403 (11.8)	9 (8.0)	627 (12.3)	8 (14.8)	
White	9626 (81.0)	84 (75.0)	4077 (80.1)	36 (66.7)	
Grade					0.709
Grade I	9373 (78.9)	33 (29.5)	4054 (79.7)	11 (20.4)	
Grade II	1700 (14.3)	15 (13.4)	708 (13.9)	7 (13.0)	
Grade III	365 (3.1)	13 (11.6)	149 (2.9)	14 (25.9)	
Grade IV	447 (3.8)	51 (45.5)	176 (3.5)	22 (40.7)	
Histology					0.316
ATC	339 (2.9)	44 (39.3)	125 (2.5)	17 (31.5)	
FTC	747 (6.3)	26 (23.2)	266 (5.2)	10 (18.5)	
MTC	93 (0.8)	2 (1.8)	45 (0.9)	2 (3.7)	
PTC	10,706 (90.1)	40 (35.7)	4651 (91.4)	25 (46.3)	
T stage					0.237
T0	5 (0.0)	1 (0.9)	1 (0.0)	0	
T1	6571 (55.3)	8 (7.1)	2901 (57.0)	7 (13.0)	
T2	2030 (17.1)	13 (11.6)	802 (15.8)	2 (3.7	
T3	2469 (20.8)	28 (25.0)	1068 (21.0)	13 (24.1)	
T4	810 (6.8)	62 (55.4)	315 (6.2)	32 (59.3)	
N stage					0.736
N0	8799 (74.0)	53 (47.3)	3783 (74.4)	23 (42.6)	
N1	3086 (26.0)	59 (52.7)	1304 (25.6)	31 (57.4)	
Laterality					0.816
Unilateral	11,815 (99.4)	111 (99.1)	5042 (99.1)	53 (98.1)	
Bilateral	70 (0.6)	1 (0.9)	45 (0.9)	1 (1.9)	
Insurance status					0.921
Insured	11,607 (97.7)	110 (98.2)	4979 (97.9)	53 (98.1)	
Uninsured	278 (2.3)	2 (1.8)	108 (2.1)	1 (1.9)	
Marital status					0.926
Married	9215 (77.5)	92 (82.1)	3901 (76.7)	48 (88.9)	
Unmarried	2670 (22.5)	20 (17.9)	1186 (23.3)	6 (11.1)	

Abbreviations: ATC, anaplastic thyroid cancer; BM, bone metastasis; FTC, follicular thyroid cancer; MTC, medullary thyroid cancer; NBM, no bone metastasis; PTC, papillary thyroid cancer.

### 2.4 Model improvement

After the first round of model building was completed, we extracted the important features from the first round of modeling process. Afterward, we adjusted the parameters of the RF model, iterated over the ntree values from 1 to 500 to choose the best ntree value (ntree = 7) (Figure 2A), and performed further model building using the extracted important features, and the model was 10‐folds cross‐validated in the training set (Figure 2B) and validated in the test set. This reduces the impact of redundant features on the model, while fewer features can improve the clinical ease of use of the model. Also, additional machine learning algorithms such as classifier (Ada), DT, Naive Bayes classification (NBC), and Support vector machine were introduced for comparison.^16^, ^17^, ^18^, ^19^, ^20^

## 3 RESULTS

### 3.1 Demographic and pathological characteristics

A total of 17,138 patients with TC were enrolled in this study. Of these patients, 166 developed bone metastases (0.97%) and 16,972 were without bone metastases (99.03%) at primary diagnosis. All patients were completely randomized in a ratio of 7:3 into a training set (*n* = 11,997) and a test set (*n* = 5141). And demographic and clinicopathological variables are detailed in Table 1.

### 3.2 Model analysis and variable influence on prediction

**FIGURE 3:**
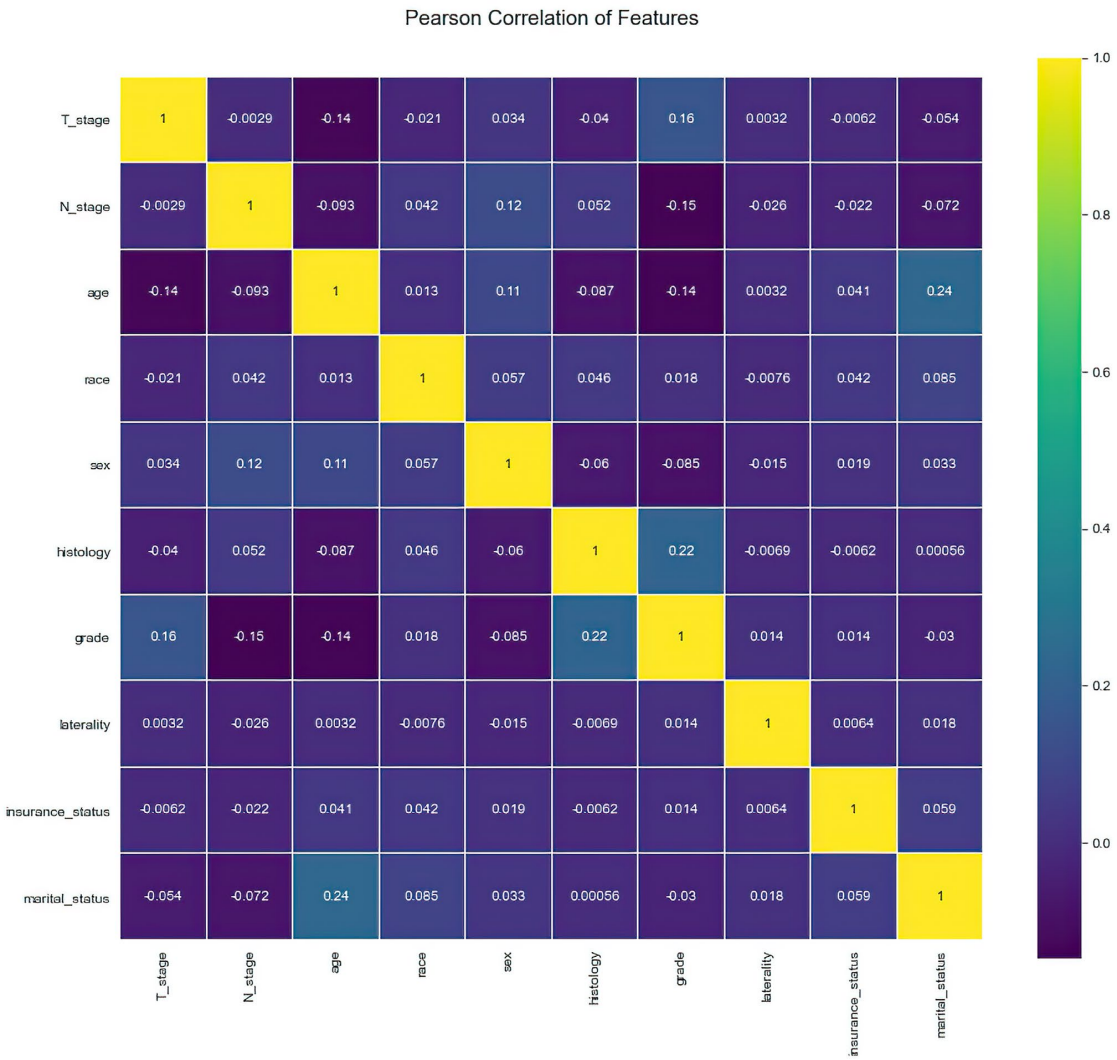
Results of Pearson correlation analysis between all variables. The heat map shows the correlation between the variables

**FIGURE 4:**
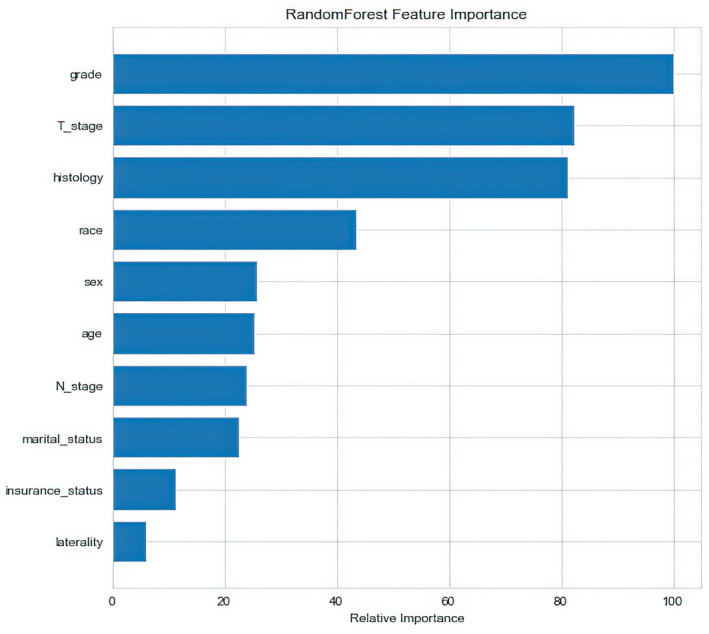
Feature importance derived from random forest model. The plot shows relative importance of the variables in random forest model

All variables were tested for Pearson correlation with each other, and the correlation heat map showed no significant correlation between them (Figure 3), indicating that the variables are independent of each other. For multivariate LR model with enter variable selection method, seven characteristics were identified as independent risk factors, including sex (*p* = 0.015), age (*p* = 0.011), race (*p* < 0.001), grade (*p* = 0.029), histology (*p* = 0.043), T stage (*p* < 0.001), and N stage (*p* = 0.005) (Table 2). For RF model, variable importance was evaluated in terms of out‐of‐bag (OOB) error rate, which can reflect the contribution of each variable when categorizing BM versus no BM (Figure 4). Grade, followed by T stage and histology were the top three most important variables. Interestingly, in the RF model, the top seven most important variables are consistent with the risk factors screened by the LR model.

**TABLE 2:** Multivariable logistic regression model with enter variable selection

Variables	OR (95% CI)	*p* value
Age		
<50	Reference	
≥50	2.045 (1.181–3.543)	0.011*
Sex		
Male	Reference	
Female	0.611 (0.411–0.908)	0.015*
Race		
Black	Reference	
Other	0.380 (0.219–0.658)	<0.001*
White	1.296 (0.638–2.633)	0.473
Grade		
Grade I	Reference	
Grade II	1.079 (0.370–3.146)	0.89
Grade III	1.713 (0.563–5.218)	0.343
Grade IV	3.318 (1.143–9.707)	0.029*
Histology		
ATC	Reference	
FTC	2.458 (1.028–5.879)	0.043*
MTC	0.928 (0.203–4.242)	0.923
PTC	0.141 (0.079–0.250)	<0.001*
T stage		
T0	Reference	
T1	0.210 (0.018–2.416)	0.211
T2	2.024 (0.948–4.319)	0.068
T3	3.090 (1.253–7.616)	0.014*
T4	8.804 (3.214–24.114)	<0.001*
N stage		
N0	Reference	
N1	1.935 (1.219–3.072)	0.005*
Laterality		
Unilateral	Reference	
Bilateral	1.287 (0.166–9.987)	0.809
Insurance status		
Insured	Reference	
Uninsured	0.700 (0.161–3.047)	0.634
Marital status		
Married	Reference	
Unmarried	0.995 (0.586–1.689)	0.985

Abbreviations: ATC, anaplastic thyroid cancer; FTC, follicular thyroid cancer; MTC, medullary thyroid cancer; PTC, papillary thyroid cancer.

* *p* < 0.05.

### 3.3 Model performance

**FIGURE 5:**
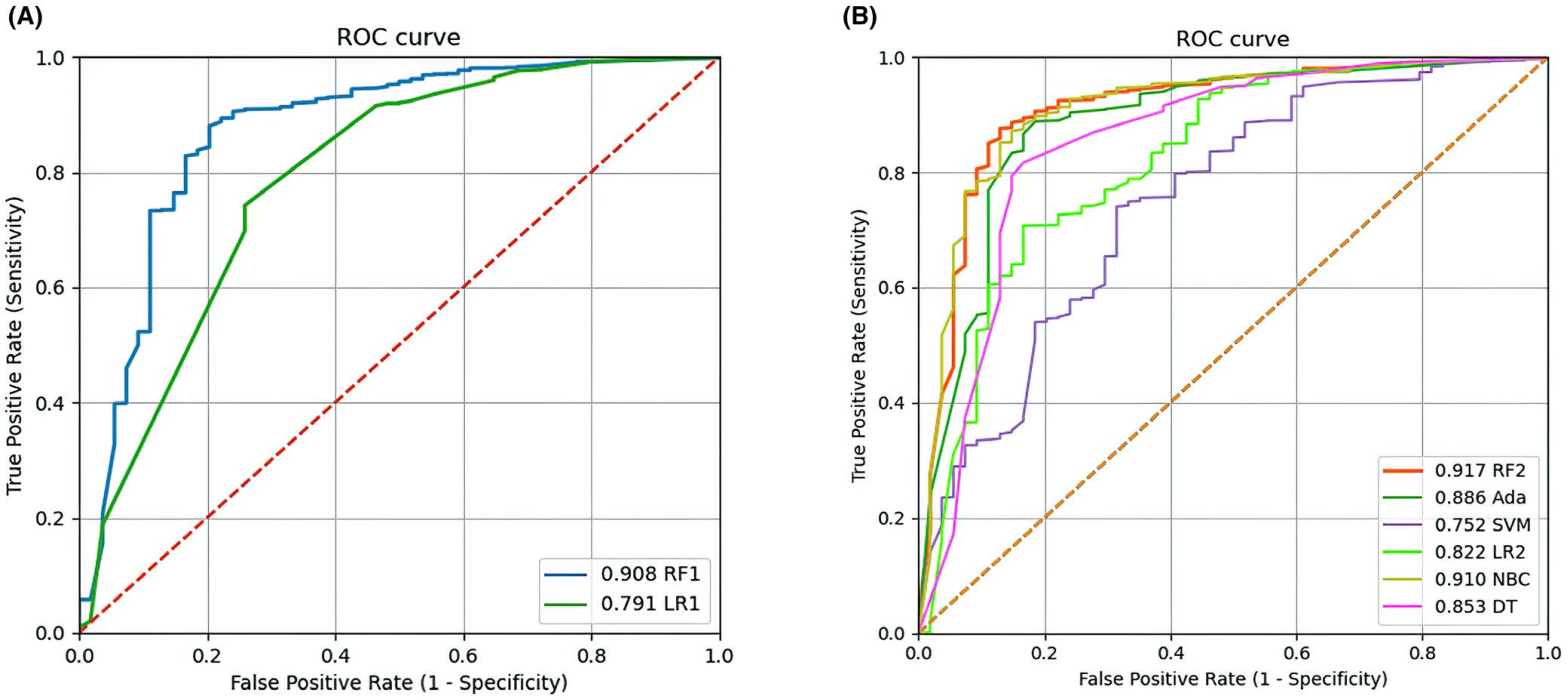
(A) The receiver operating characteristic (ROC) curve of the initial random forest (RF1) model and initial logistic regression (LR1) model. (B) The ROC curve of different improved machine learning models

The test set was applied to test and compare the predictive performance of the all models. The AUC, accuracy score, recall rate, and specificity were used to evaluate and compare the model performances. The initial random forest (RF1) model performs better than the initial logistic regression (LR1) model (AUC: 0.908, accuracy: 0.877, sensitivity: 0.796, specificity: 0.878 vs. AUC: 0.791, accuracy: 0.743, sensitivity: 0.741, specificity: 0.742, Table 3; Figure 5A). After that, we adjusted the parameters of the RF model and iterated over the ntree values from 1 to 500 to choose the ntree value that makes the best prediction performance (ntree = 7, Figure 2A). The improved random forest (RF2) model using the top seven significant features has the best prediction performance among all machine learning models (AUC: 0.917, accuracy: 0.904, sensitivity: 0.833, specificity: 0.905, Table 3; Figure 5B). It also achieved excellent performance in the 10‐fold cross‐validation of the training set (average AUC = 0.916, Figure 2B). Meanwhile, the prediction results of the improved RF model are shown in Table 4, which intuitively shows its prediction power.

**TABLE 3:** Comparison prediction performances of different models for BM

Models	AUC	Accuracy	Recall rate (sensitivity)	Specificity
Initial				
LR1	0.791	0.743	0.741	0.742
RF1	0.908	0.877	0.796	0.878
Improved				
Ada	0.886	0.887	0.812	0.888
DT	0.853	0.817	0.833	0.816
LR2	0.822	0.708	0.833	0.707
NBC	0.910	0.871	0.852	0.871
RF2	0.917	0.904	0.833	0.905
SVM	0.752	0.739	0.685	0.740

Abbreviations: Ada, AdaBoost classifier; AUC, area under the curve; DT, decision tree; LR1, initial logistic regression; LR2, logistic regression improved; NBC, Naive Bayes classification; RF1, Initial random forest; RF2, Random forest improved; SVM, support vector machine.

**TABLE 4:** Prediction results of the improved random forest model

Predictive	Actual	
	BM	NBM
BM	45 (TP)	486 (FP)
NBM	9 (FN)	4601 (TN)

Abbreviations: BM, bone metastasis; FN, false negative cases; FP, false positive cases; NBM, no bone metastasis; TN, true negative cases; TP, true positive cases.

## 4 DISCUSSION

Bone metastases can cause severe spinal cord compression, pathologic fractures, bone pain, and other SREs, thus, worsening the patient's life quality. It has been reported that approximately 78% of patients with BM from TC developed at least one SRE.^6^ A research^21^ observed 52 BM patients out of 1398 DTC patients (3.7%). Similar results were reported in a study 3 years ago, in which 3.9% (1173) of TC patients developed BM.^8^ In the present study, the prevalence of BM in patients with TC was less than previously reported, only 0.97%. This may be due to the fact that the data recorded in the SEER database were diagnostic of BM at the same time, whereas the BM data in the other studies were cumulative data at different times. So the incidence of BM was lower in this study. From the above, it can be seen that in patients with TC, the probability of developing a BM at the primary diagnosis is low, and most BMs develop during the clinical follow‐up after the initial diagnosis of TC. Therefore, after the initial diagnosis of TC patients, further follow‐up examination of those patients with a high probability of developing bone metastases is important for receiving appropriate treatment and improving prognosis. Bone scintigraphy is usually used to identify possible bone metastases in patients newly diagnosed with TC. However, because bone scintigraphy is expensive and has radiation damage, further follow‐up examination may not be appropriate with this method. Pathological diagnosis is considered the gold standard. However, studies have shown that biopsy is not only difficult and painful, but also increases the risk of tumor cell proliferation, which means it may not be safe for routine diagnosis.^22^ To better address this problem, we used advanced machine learning algorithms and constructed a RF model to identify BM high‐risk TC patients.

Random forest seems to be the machine learning algorithm of choice in most clinical studies.^23^, ^24^ Studies have shown that it is one of the most accurate machine learning models, and is superior to other techniques in handling large numbers of features and highly nonlinear data, is agile in handling data noise, and is easier to tune and integrate with learning algorithms than other algorithms.^25^ In the research, we found that advanced machine learning techniques like RF modeling can improve the utilization of information in analytical databases and enable the development and validation of predictive models with better performance. The RF model has stronger predictive performance, probably because the RF model uses more advanced classification decisions and different weighting ratios compared to the other model. The model has shown excellent performance in predicting BM in TC patients, which can provide clinicians with more accurate and personalized health‐care decisions. The potential use of this model is to help patients with TC predict the likelihood of bone metastases and to alert patients at high risk of BM for further investigation, which may help improve their prognosis.

In this study, we found that the top seven most important features in the RF model are precisely the risk factors screened out in the LR model, including grade, T stage, histology, race, sex, age, and N stage. Although SRE has long been recognized as a sign of BM, it is not reasonable to consider targeted screening for BM in TC patients only when they have symptoms of bone involvement, as this would delay their treatment. Therefore, models are necessary to predict patients with TC at high risk for bone metastases, and to provide early attention and screening. In previous studies,^26^, ^27^, ^28^, ^29^ age has been demonstrated to have an impact on the prognosis of TC patients, and it has been reported that the risk of DM was significantly reduced in younger TC patients compared with older patients.^30^ And we found that age was also an important feature influencing BM in our study. Zhao et al.^31^ found that sex was a risk factor for TC lateral lymph node metastasis and skip metastasis. In this research, we also found that sex is an important characteristic that affects BM, with men being more likely to develop BM than women.

There are now many studies shows that tumor biology is believed to play an important role in disease development, which may be closely related to the occurrence and development of BM. A meta‐analysis found significant correlations between tumor multifocality, size, vascular infiltration, extrathyroidal extension, and lymph node metastasis and DM.^32^ In the present study, we found that T and N stage were important features predicting the development of bone metastases in patients with TC. This study also found that patients with poorly or undifferentiated tumors were more likely to develop BM, possibly because cancer cells invade surrounding tissues, capillaries, and lymphatic vessels, and these poorly or undifferentiated tissues have a greater potential to grow and undergo early metastasis. These findings are consistent with those of Sugino et al.^33^ Thyroid cancer is highly heterogeneous in terms of clinical and molecular characteristics and consists of four major subtypes associated with different propensities of BM. In this study, PTC was the most common type of TC, but FTC was more likely to develop BM, which is consistent with the findings of Do et al.^34^ This may be because vascular invasion in FTC is more common and reasonable than vascular invasion in PTC.

This study applied machine learn‐based RF methods with SEER data to predict BM in TC patients. It extends the LR‐based nomogram model that has been used frequently by other researchers recently. However, this study still has several limitations. First, the model is based on machine learning and deep learning algorithms, so there may be some difficulties in clinical interpretation of the important features screened out by the model. Second, this is a study based on a North American population, so there may be gaps in population applicability, so it is necessary to include a broader population in future studies. Third, the SEER database records information at the time of initial diagnosis, which means that subsequent treatment data are missing, and we were unable to include them in the BM prediction analysis of TC patients.

## 5 CONCLUSION

In conclusion, here, we developed a RF prediction model for bone metastases in TC patients that outperformed traditional LR models. This facilitates personalized diagnosis and refined clinical decision making for BM in TC patients.

## CONFLICT OF INTEREST

No benefits in any form have been or will be received from any commercial party related to the subject of this manuscript.

## ETHICS APPROVAL AND CONSENT TO PARTICIPATE

We received permission to access the research data file in the SEER program from the National Cancer Institute, US. Approval was waived by the local ethics committee, as SEER data is publicly available and de‐identified.

## Data Availability

The data sets generated and/or analyzed during the current study are available in the SEER database (https://seer.cancer.gov/).
